# New Miniaturized Disposable Screen-Printed Microchip Integrated with Molecularly Imprinted Polymer for Metronidazole Benzoate Drug Detection

**DOI:** 10.3390/mi13122107

**Published:** 2022-11-29

**Authors:** Menna El-Beshlawy, Hassan Arida

**Affiliations:** 1 Department of Chemistry, Faculty of Women, Ain Shams University, Cairo 11566, Egypt; 2 Department of Pharmaceutical Chemistry, College of Pharmacy, Taif University, P.O. Box 11099, Taif 21944, Saudi Arabia

**Keywords:** screen-printed microchip, molecularly imprinted polymer, drug detection, metronidazole benzoate

## Abstract

A novel potentiometric microelectrode incorporating a molecularly imprinted polymer (MIP) was fabricated, characterized, and successfully applied to the recognition and quantification of the drug, metronidazole benzoate. The elaborated MIP-based sensor was realized by thermal polarization, using metronidazole benzoate as the template material, 1-vinyl-2-pyrrolidine (VP) as a functional monomer, and ethylene glycol dimethacrylate (EGDMA) as the cross-linking agent in the presence of benzoyl peroxide as the initiator. The MIP-based sensor exhibited a super-Nernstian response (61.5 ± 0.5, mV/decade) covering the linear concentration range of 1 × 10^−8^–1 × 10^−3^ mole L^−1^ of metronidazole benzoate with a fast response time (≤10, s.) and detection limit of 7 × 10^−9^ mole L^−1^. The microchip showed high selectivity toward the template drug molecule in the presence of many investigated interfering species. The chip electrode was successfully used in the quantification of metronidazole benzoate in some real biological samples with high accuracy (recovery, 95.4%) and precision (RSD, 1.5). Moreover, the merits offered by the elaborated MIP-based MB microchip assembly include small size, miniaturization, integration, and consequently, automation feasibility.

## 1. Introduction

Metronidazole benzoate (benzoyl metronidazole), 2-(2-Methyl-5-nitro-1H-imidazol-1-yl) ethyl benzoate (MB), belongs to the category of antiprotozoal drugs and is mostly used as an antibacterial agent prescribed against different diseases including, colonic, periodontitis and many other anaerobic infections. It has been widely used due to being tasteless and having greater palatability with fewer doses required compared to other antibiotics. Metronidazole benzoate has 98–100% bioavailability with a 7–8 h. half-life and hepatic metabolism [1,2,3]. Several methods have been demonstrated in the literature for the quantification of (MB) in pharmaceutical formulations and biological samples due to the vital importance of the drug [4,5,6,7,8,9,10,11,12,13,14]. The different analytical protocols currently used for the quantification of (MB) drugs include spectrophotometric techniques [4,5,6,7], capillary electrophoresis [8], voltammetric methods [9], different chromatographic tools [10,11,12], and bulk selective potentiometric electrodes [13]. However, the use of these methods involves sophisticated devices and tedious steps in sample preparations, and also requires expensive instrumentation. These factors increase the cost and time of analysis and also limit the applicability of such instrumental tools for a large number of samples [5]. Moreover, accurate, precise, and fast analysis methods for MB still represent a challenge and hold a great significant interest in chemical analysis for many scientists.

On the other hand, microchip-based potentiometric sensors, particularly screen-printed microchips, have been attracting increasing attention in environmental, clinical, and pharmaceutical analysis. These disposable micron-scale chips with small sample volumes can gently manipulate fluid flow thus providing more efficient and alternative tools in a broad range of applications, particularly in drug analysis. These microsensors have been advanced by their simple operation, high throughput, parallel analysis, and quick response leading to overall reduced costs, and making it much easier to elaborate product transfer in practical applications [14,15,16,17,18,19,20,21,22,23].

Moreover, a molecularly imprinted polymer (MIP) was recently applied as a sensitive element in the fabrication of potentiometric electrodes [13,24,25,26,27,28,29,30,31,32,33]. MIP-sensitive elements represent novel materials and have appeared to be very promising candidates as highly selective membranes in potentiometric applications where analyte selectivity is crucial. These materials are mainly prepared by the polymerization of functional monomers in the presence of a template molecule, particularly drug species. The template drug species is leached out leaving behind pores that are integral in size, functionality, and shape, to the drug species. Recently, these MIPs yielded superior selectivity and consequently developed increased applicability in analytical chemistry, namely catalytic control, purification of racemic mixtures, and chemical sensing of complex species particularly drug species [8,13]. In this context, MIP materials have been used as a sensitive membrane in the fabrication of chemical sensors which have been successfully applied for the determination of illegal drugs and additives [26], lansoprazole detection [27], ivabradine hydrochloride quantifications [28], measurements of pharmaceutical and biological species [29], drug analysis [30], biomolecule sensing design to intraoral fluid testing [31], electrochemical sensors and electronic tongues [32], and the analysis of a veterinary drug, imidocarb dipropionate [33].

To the best of our knowledge, there have never been microsensors nor MIP chips reported for the detection of the drug, metronidazole benzoate. In this study, an attempt was made to realize a novel, simple, rapid, sensitive, cost-effective MIP-based screen-printed microchip for the detection of metronidazole benzoate with good accuracy and precision in some real biological samples.

## 2. Materials and Methods

### 2.1. Reagents and Chemicals

All used chemicals and reagents were of “analytical” reagent grade unless otherwise stated. Furthermore, deionized distilled water was used to wash the glassware and in the preparation of the reagents, throughout. Nitrate or chloride salts of the metal used in the studies were purchased from Riedel-de Haën (Riedel-de Haën, Seelze, Germany). Disposable screen-printed microchip carbon electrodes (0.25 mm PET, 3 mm/6 mm in diameter, graphene-modified SPE) were obtained from Suzhou Delta-biotech (Ltd, Suzhou, China) and used as the microelectrode substrate. A purified multiwall carbon nanotube (id: 5–12 nm, od: 30–50 nm, length: 10–20 µm, purity: >95%,) was purchased from Chengdu organic chemicals Company “COCC”, China (“COCC”, Chengdu, China). Potassium Tetrakis (4-chlorophenyl) borate lipophilic additive and solvent mediator, 2-nitrophenyl octyl ether were purchased from Sigma-Aldrich (CH-9471 Buchs, Buchs, Switzerland). Tetrahydrofuran (THF) and high molecular weight carboxylated poly (vinyl chloride) (220,000, PVC) were purchased from Riedel-de Haën Chemical Company (Riedel-de Haën Chemical Company, Seelze, Germany). Metronidazole benzoate drug raw material (purity: 99.5 %) was obtained from the Egyptian FDA (The Egyptian FDA, Cairo, Egypt). Glycine, Citric acid, Barbituric acid, and D-Glutamic acid, were purchased from Dr. Ehrenstorfer GmbH (Berlin, Germany). Polyaniline (emeraldine salt) (3–100 μm particle size, average Mw > 15,000), ethylene glycol dimethacrylate (EGDMA), 1-vinyl-2-pyrrolidine (VP), benzoyl peroxide (BPO), D-glucose, acetic acid, phosphoric acid, and acetonitrile were purchased from Sigma-Aldrich Inc. (Sigma-Aldrich Inc, St. Louis, MO, USA).

### 2.2. Microfabrication of MIP-Based MB Chip Assembly

A molecularly imprinted polymer (MIP)-sensitive element was synthesized utilizing the precipitation polymerization methodology [25,26,27,28,29,30,31,32,33]. In such a method, MIP-based beads were obtained by incorporating 1.0 mmol metronidazole benzoate as a template drug molecule with 3.0 mmol of 1-vinyl-2-pyrrolidine (VP) monomer (Riedel-de Haën, Sleeze, Germany), 3.0 mmol of ethylene glycol dimethacrylate (EGDMA) (Riedel-de Haën, Sleeze, Germany)as a cross-linking agent and 60.0 mg of benzoyl peroxide (BPO) (Riedel-de Haën, Sleeze, Germany) as initiator. The mixture was properly dissolved in 20 mL acetonitrile and transferred to a 30-mL glass-capped bottle. The mixture was then purged with a flow of N_2_ gas for 10 min to remove the dissolved oxygen gas and homogenized by sonication for 15 min in a sonicator. The glass-capped container containing the mixture was then, heated at 80 °C for 18 h in an oil bath to complete the polymerization process. The template drug molecules were eliminated by Soxhlet extraction using a NaOH/methanol mixture (1:9, *v/v*). The obtained polymer beads were cleaned by washing them with methanol and then left to dry overnight at room temperature. For a comparison study, a nonimprinted polymer (NIP) was also synthesized in the absence of drug template molecules and under typical circumstances.

Prior to fabrication of the microsensor, the screen-printed microchip substrate was sonicated for 5 min., cleaned by soaking in ethanol for an additional 10 min. and left to dry for 1 h. at room temperature. A drop-casting technique was used to apply an aliquot of 10 µL of 2 mg/mL PANI onto the conductive screen-printed graphene microchip substrate and the resulting conductive polymer layer was left to dry for 1 h. at room temperature.

The cocktail coating mixture was prepared by thoroughly mixing 10 (5.0 wt.%) mg of the MIP-sensing element, 66 mg (33.0 wt.%) of PVC, 120 mg (60.0 wt.%) of DNPOE plasticizer, and 4 mg (2.0 wt.%) of MWCNT in 2.0 mL of THF solvent. The electrode assembly responsive to metronidazole benzoate was micro-fabricated by nebulizing the cocktail coating mixture onto the surface of the screen-printed microchip substrate, as described recently in our previous work [18]. The realized electrode assembly was soaked in 10^−2^ mol L^−1^ metronidazole benzoate solution for 1 h. before measurements and stored dry in air when not in use.

The elaborated microchip electrode was characterized at room temperature (25 ± 1 °C) as a metronidazole benzoate sensor by measuring the electromotive force (EMF) of the microchip in conjunction with an Orion single junction reference electrode using a pH/mV meter (Orion 720/SA, Cambridge, MA, USA) after the successive immersion of the electrode assembly in the stirred metronidazole benzoate standard calibration solutions. While the performance characteristics of the electrode assemblies were assessed in accordance with the IUPAC standards, all trials were performed three times at a minimum. 

## 3. Results and Discussion

### 3.1. Characterization of the MIP-Based MB Microchip

In order to develop an accurate, precise, and reliable method for metronidazole benzoate analysis, a disposable screen-printed microchip based on a molecularly printed polymer was fabricated, characterized, and applied in the determination of the drug in some real biological samples. For this purpose, many trials were performed using different membrane compositions with different concentrations and without some additives namely potassium tetrakis (4-chlorophenyl) borate, MWCNTs, and PANI (unpublished data). The combination of PANI with the sensitive membrane and incorporating 10 mg of the MIP-sensing element, 66 mg of PVC, 120 mg of DNPOE plasticizer, and 4 mg of MWCNT offered the best potentiometric response parameters toward metronidazole benzoate detection. The reset of characterization and application experiments were, therefore, performed using a microchip fabricated from this combination.

**Figure 1:**
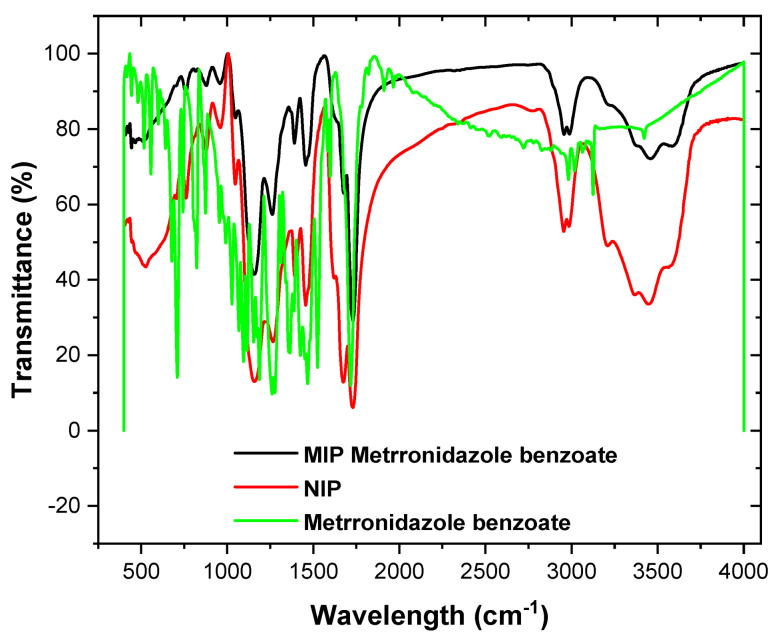
FT-IR spectra of the MIP, NIP, and MB drug template materials.

Fourier-transform infrared) FT-IR (spectroscopy (Brucker, Billerica, MA, USA) studies were conducted for the MIP metronidazole benzoate, NIP, and metronidazole benzoate template, and the results obtained are presented in Figure 1 While the three substances offered different spectra, the hydrogen bond formed between the imprinted polymer and the drug template appears in the spectra and clearly provides evidence for the interactions between the two molecules [33]. Specifically, the MIP and NIP FT-IR spectra showed a strong and broad peak at 3440 and 3448 cm^−1^ for νOH stretching and other peaks at 1690 and 1730 cm^−1^ for νC=O stretching, respectively, while the MIP spectrum also showed a broad O–H stretching peak at 3440 cm^−1^, and CH stretching at 2967 cm^−1^.

The microsensor based on the MIP modified by multiwall carbon nanotubes was realized by nebulization of the sensitive membrane coating mixture onto the surface of the square planer screen-printed microchip substrate using a new methodology recently developed [18]. The combination of the MIP technology modified by MWCNT with screen-printed microchip tools realizes very interesting, miniaturized, and new generations of potentiometric microsensors responsive to biological and drug species. These microdevices offer the merits of high sensitivity, reasonable selectivity, simple construction, fast response, mass production, small size, cost-effective, versatile applications, and integration and automation feasibility.

**Figure 2:**
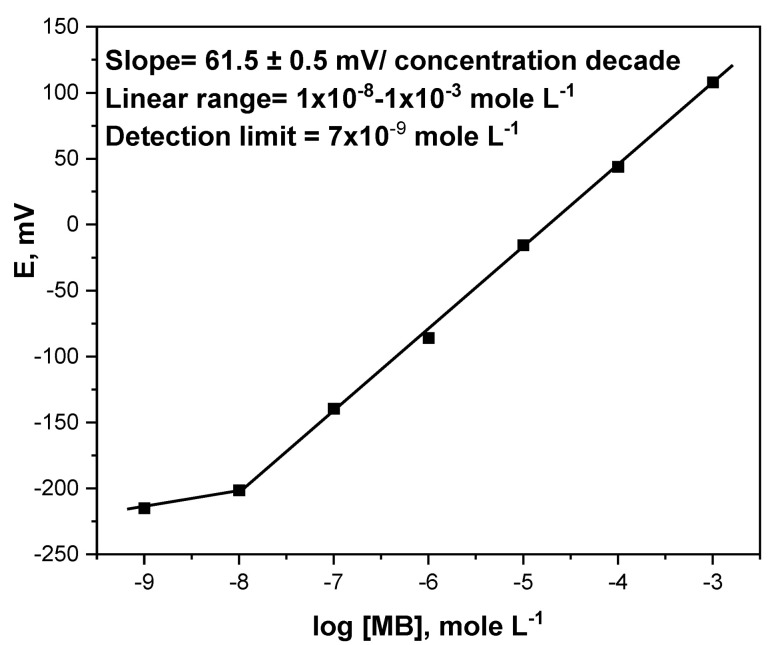
Potentiometric calibration plot of the MIP-based MB microchip.

Three assemblies of the realized MIP-based MB microchip were fabricated and calibrated in triplicates as metronidazole benzoate electrodes and the results obtained are presented in Figure 2. This microchip provides a super-Nernstian behavior toward MB drugs with high sensitivity (61.5 ± 0.5 mV/concentration decade) and covers the linear range of 1 × 10^−8^–1 × 10^−3^ mole L^−1^ with a detection limit of 7 × 10^−9^ mole L^−1^. The superior potentiometric sensitivity of the realized microchip is attributed to the combination of the MIP, MWCNT, and screen-printed substrate [13,18]. While the CNTs showed high ion-exchange properties and excellent conductivity because they have a high surface-to-volume ratio, the screen-printed square planer substrate dramatically enhances the sensitivity of the potentiometric electrode [18].

**Figure 3:**
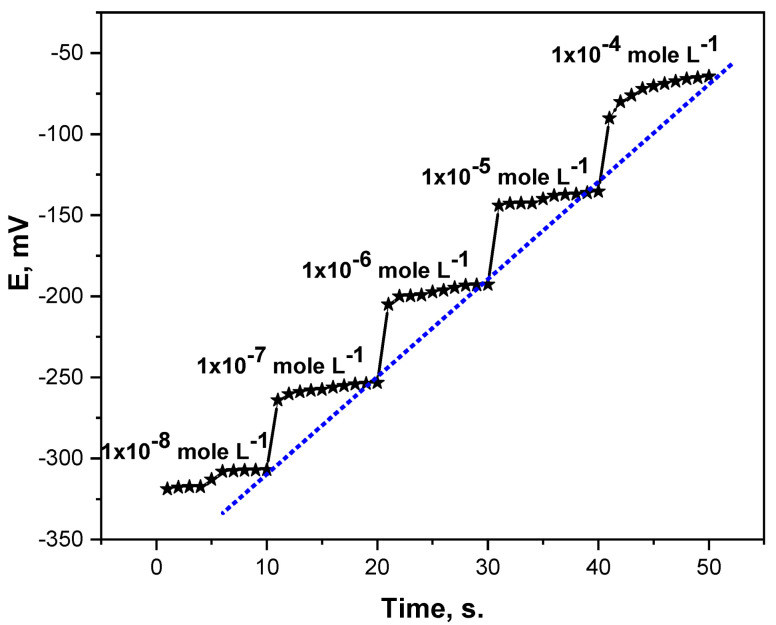
Potentiometric dynamic response of the MIP-based MB microchip.

The potentiometric dynamic response of the elaborated MIP-based MB microsensor was investigated by successive immersing of the microchip in a series of MB drug standard solutions of 1 × 10^−8^–1 × 10^−4^ mole L^−1^ and recording the corresponding potential at constant time intervals. The results obtained (Figure 3) showed that the MB microchip provides a very short response time (10 s.) in the tested linear range. Moreover, the long lifetime of the microchip was assessed by frequent calibration of the microchip using freshly prepared metronidazole benzoate drug solutions. The data obtained showed that the microchip offered a relatively long lifespan (≥4 months).

**Figure 4:**
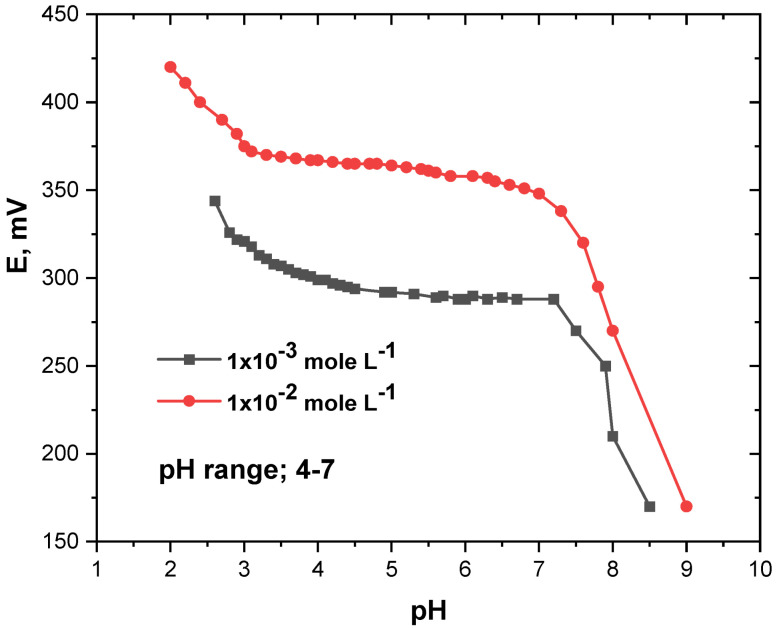
Influence of the pH on the response of the MIP-based MB microchip.

The influence of the pH of the test solutions on the potentiometric response of the elaborated microchip assembly was investigated using two MB drug solutions (1 × 10^−3^ and 1 × 10^−4^ mole L^−1^). For this purpose, small aliquots of concentrated nitric acid and sodium hydroxide were used to change the pH of the test solutions and the change in the potential of the chip was recorded versus pH. The results obtained are presented in Figure 4. As can be seen, the potential of the MB microchip was almost constant with the change in the pH of the test solutions in the pH range of 4–7 and consequently, this pH range of the test solution was used in all characterization and application studies conducted on the microchip. The higher and lower potential responses of the chip noticed at acidic and basic media, respectively, were attributed to the deterioration of the sensitive membrane coat and the change in the membrane phase in highly acidic and basic solutions.

**Figure 5:**
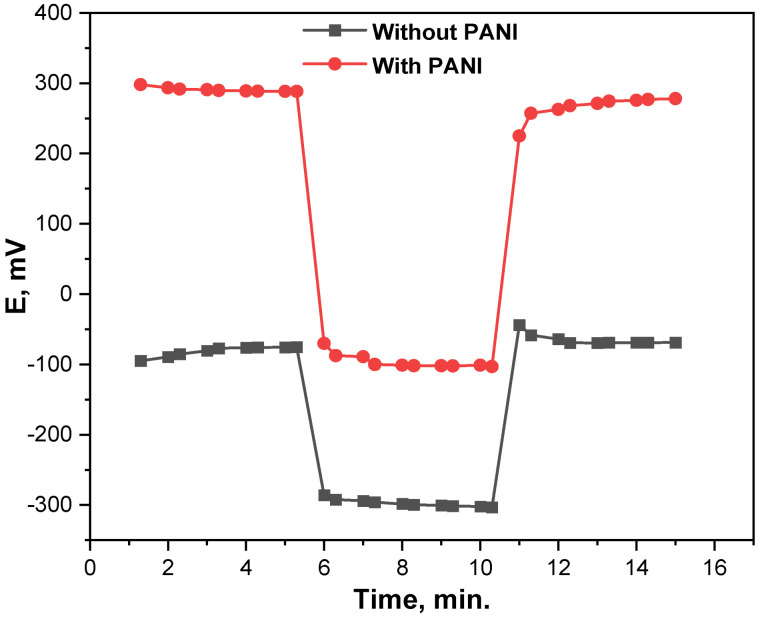
Layer effect on the response of the MIP-based MB microchip (10^−4^ mole L^−1^) with and without PANI.

Two assemblies of the MIP-based MB microchip fabricated with and without PANI have been used in the study of the water layer effect on the potentiometric response of the elaborated microsensor. In this study, the potential of the chip versus time intervals was recorded after successive immersing of the chip in water followed by the immersing of the chip in 10^−4^ mole L^−1^ of MB drug solution. The results obtained are depicted in Figure 5 and provided a complete lack of potential drift of the potentiometric response of the realized microelectrode. The elaborated microchip assembly fabricated with PANI offered a fast equilibrium, stable behavior, and consequently high stability and reliability toward MB. These improvements in the response parameters were attributed to the presence of the PANI film which was injected between the sensing membrane and the surface of the screen-printed graphene. PANI was successfully used as a conducting polymer to create solid contacts between an electronic conductor substrate and an organic sensitive membrane with high conduction, great stability, and hydrophilicity of the surface [29]. Furthermore, the nebulization methodology of the sensitive membrane coating mixture recently developed significantly increases membrane stability and consequently improves the chip potentiometric response parameters [18].

**Figure 6:**
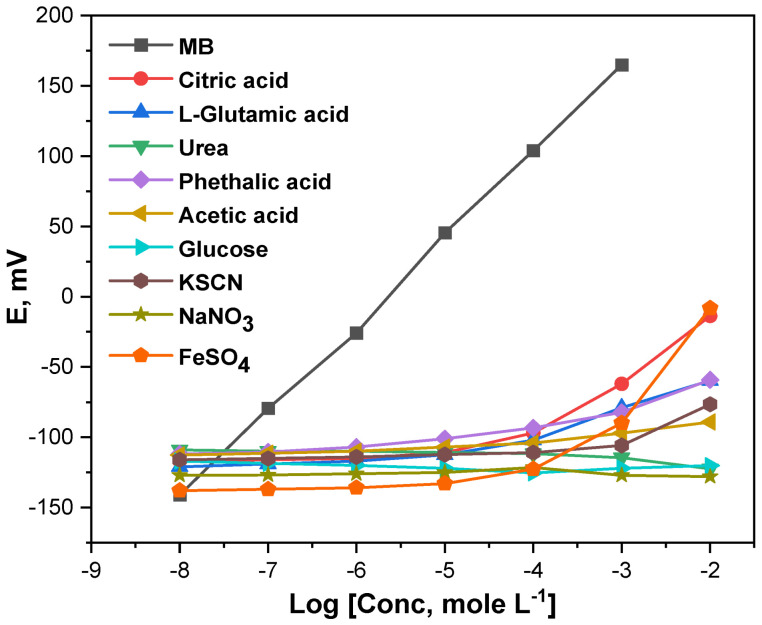
Potentiometric response of the MIP-based microchip toward MB and some interference species.

The influence of the potential interference on the electrochemical behavior of the MIP-based MB microchip was investigated. In this study, the MIP-based MB microchip was calibrated from the MB drug and some potential interference species, and the results obtained are presented in Figure 6. As can be seen, the MIP-based MB microchip showed reasonably high selectivity toward MB compared to the other tested species. A similar high selectivity was reported for the MIP-based ion selective electrodes and potentiometric sensors which is attributed to the MIP possessing a unique property of recognizing specific template molecules [24].

The data obtained from the potentiometric response parameters of the elaborated MIP-based MB screen-printed microchip are summarized in Table 1. The new MIP-based MB microchip provided high sensitivity (61.5 ± 0.5, mV/decade) toward MB covering the linear range of 1 × 10^−8^–1 × 10^−3^ mole L^−1^ with high selectivity, the fast response time (≤10, s.) and a long lifespan (≥4, months).

### 3.2. Analytical Applications of the MIP-Based MB Microchip

Based on the obtained results, the novel, elaborated, MIP-based MB microchip was successfully applied in the determination of the tested drug (MB) in some real urine samples. In this study, 50 mL of morning urine samples were collected from three male volunteers of ~20 years old. A known amount of pure MB was injected into each urine sample and was frequently determined by the newly realized microchip. Each sample was determined in triplicates using a freshly performed calibration plot of the MB chip and the accuracy values were calculated. The results obtained are summarized in Table 2. The average recovery obtained was 95.4 % and the RSD was 1.5.

## 4. Conclusions

The screen-printed chip based on a molecularly imprinted polymer (MIP) with metronidazole benzoate (MB) drug as a template and multiwall carbon nanotubes (MWCNTs) as a modifier has been micro-fabricated and characterized for selective recognition and determination of the MB molecule. The elaborated microchip revealed high sensitivity (61.5 ± 0.5 mV/decade) and reasonable selectivity toward MB covering a wide linear range (1 × 10^−8^–1 × 10^−3^ mole L^−1^) with a fast response time (≤10 s.) and long lifetime (≥4 months). The realized microchip has been successfully applied in the determination of MB in some real biological samples with high accuracy (recovery, 95.4 %) and high precision (RSD, 1.5). Further, the merits offered by the elaborated MIP-based MB microchip assembly include small size, miniaturization, integration, and consequently, automation feasibility.

## Figures and Tables

**Table 1:** Potentiometric response parameters of the MIP-based MB microchip.

Parameters	MB Microchip
Linear range, mole L^−1^	1 × 10^−8^–1 × 10^−3^
Slope, mV/decade	61.5 ± 0.5
Detection Limit, mole L^−1^	7 × 10^−9^
Lower limit of linear range, mole L^−1^	1 × 10^−8^
pH range	4–7
Lifetime, months	≥4
Response time, s.	≤10

**Table 2:** The accuracy studies of the MIP-based MB microchip (µg/10 mL urine).

No.	Injected,	Found	Recovery, %
1	275.00	265.00	96.4
2	27.50	26.17	95.2
3	2.75	2.60	94.5
Average recovery			95.4

## Data Availability

This article does not contain any additional data.
